# Concordant Signaling Pathways Produced by Pesticide Exposure in Mice Correspond to Pathways Identified in Human Parkinson's Disease

**DOI:** 10.1371/journal.pone.0036191

**Published:** 2012-05-01

**Authors:** Seema Gollamudi, Ashu Johri, Noel Y. Calingasan, Lichuan Yang, Olivier Elemento, M. Flint Beal

**Affiliations:** 1 Department of Neurology and Neuroscience, Weill Medical College of Cornell University, New York, New York, United States of America; 2 Institute for Computational Biomedicine, Weill Medical College of Cornell University, New York, New York, United States of America; Centre Hospitalier Universitaire Vaudois (CHUV), Switzerland

## Abstract

Parkinson's disease (PD) is a neurodegenerative disease in which the etiology of 90 percent of the patients is unknown. Pesticide exposure is a major risk factor for PD, and paraquat (PQ), pyridaben (PY) and maneb (MN) are amongst the most widely used pesticides. We studied mRNA expression using transcriptome sequencing (RNA-Seq) in the ventral midbrain (VMB) and striatum (STR) of PQ, PY and paraquat+maneb (MNPQ) treated mice, followed by pathway analysis. We found concordance of signaling pathways between the three pesticide models in both the VMB and STR as well as concordance in these two brain areas. The concordant signaling pathways with relevance to PD pathogenesis were e.g. axonal guidance signaling, Wnt/β-catenin signaling, as well as pathways not previously linked to PD, e.g. basal cell carcinoma, human embryonic stem cell pluripotency and role of macrophages, fibroblasts and endothelial cells in rheumatoid arthritis. Human PD pathways previously identified by expression analysis, concordant with VMB pathways identified in our study were axonal guidance signaling, Wnt/β-catenin signaling, IL-6 signaling, ephrin receptor signaling, TGF-β signaling, PPAR signaling and G-protein coupled receptor signaling. Human PD pathways concordant with the STR pathways in our study were Wnt/β-catenin signaling, axonal guidance signaling and G-protein coupled receptor signaling. Peroxisome proliferator activated receptor delta (*Ppard*) and G-Protein Coupled Receptors (GPCRs) were common genes in VMB and STR identified by network analysis. In conclusion, the pesticides PQ, PY and MNPQ elicit common signaling pathways in the VMB and STR in mice, which are concordant with known signaling pathways identified in human PD, suggesting that these pathways contribute to the pathogenesis of idiopathic PD. The analysis of these networks and pathways may therefore lead to improved understanding of disease pathogenesis, and potential novel therapeutic targets.

## Introduction

Parkinson's disease (PD) is the second most common neurodegenerative disease. It is characterized by tremor, rigidity, slowness of voluntary movement and postural instability. The motor symptoms of PD result from loss of dopaminergic (DA) neurons in the substantia nigra (SN). Intracytoplasmic eosinophilic inclusions composed of α-synuclein, are found in lewy bodies in the remaining, intact nigral neurons [1]. Monogenic mutations account for less than ten percent of all PD cases and the majority of cases are sporadic [2], [3]. As the etiology of the majority of human PD cases is unknown, several neurotoxicant models of PD have been developed to correctly model PD [4], [5]. Evidence for an environmental basis of PD comes from epidemiological studies and experimental animals exposed to environmental toxins [6] suggesting an important role of pesticides. Pesticides are defined as any substance or mixture intended for preventing, destroying, repelling or mitigating pests.

Many commonly used pesticides cause mitochondrial dysfunction and oxidative damage, similar to that observed in idiopathic PD [7]–[9]. Paraquat (PQ) is the third most commonly used herbicide in the world, and was identified as a neurotoxicant based on structural similarities to MPP^+^ (1-methyl-4-phenylpyridine), the active metabolite of 1-methyl-4-phenyl-1,2,3,6-tetrahydropyridine (MPTP). PQ is a redox cycler, pyridaben (PY) inhibits mitochondrial complex I [8], and maneb (MN) inhibits mitochondrial complex III [10]. Exposure to PQ or MN or their co-exposure causes loss of DA neurons in animal models and MN exacerbates the toxicity of PQ in mice [11]. Repeated PQ injections produce selective loss of nigral DA neurons, and α-synuclein aggregation within neurons of the SN pars compacta [12].

In rural environments, where workers were co-exposed to PQ and MN several studies have clearly shown a marked increase in Parkinson's type neurodegeneration [13]. In the present experiments, we examined the effects of pesticide exposure on gene expression. We administered the pesticides by subcutaneous alzet pumps and sacrificed mice at day 7. We believe the use of osmotic pump is advantageous because it will deliver pesticide at a constant rate as opposed to the episodic peaks in levels observed with intra-peritoneal (i.p) injections.

Prior studies of gene expression profiling have been done in postmortem brain samples from human PD patients [14]–[17]. Consensus pathways were identified in a cross comparison of whole genome expression profiles using standardized pathway analysis [18] and compared to genome wide association studies [19]. Prior *in vivo* studies examined gene expression profiling in PQ and MN+PQ treated mice, but there are no reports on PY alone [20], [21].

Exposure to pesticides may elicit alterations in signaling pathways in the VMB and STR, which may be useful in identifying targets for therapeutic intervention. In the present study, after showing that pesticide treatment induces consistent cell death of DA neurons in the SN, we used RNA-Seq next generation sequencing technology to comprehensively quantify expression levels of all known genes and isoforms in treated versus untreated animals. We then examined them by standardized pathway analysis using Ingenuity Pathway Analysis (IPA). We examined samples of VMB and STR which then allowed assessment of the involvement of both non neuronal and neuronal populations in pesticide models of PD. We observed that PQ, PY and MNPQ elicit concordant signaling pathways in the VMB and STR, which concur with pathways obtained from human PD gene expression profiling, and genome wide association studies.

## Results

### Pesticide Induced Loss of TH+ Neurons and Increased α-synuclein Aggregates

**Figure 1:**
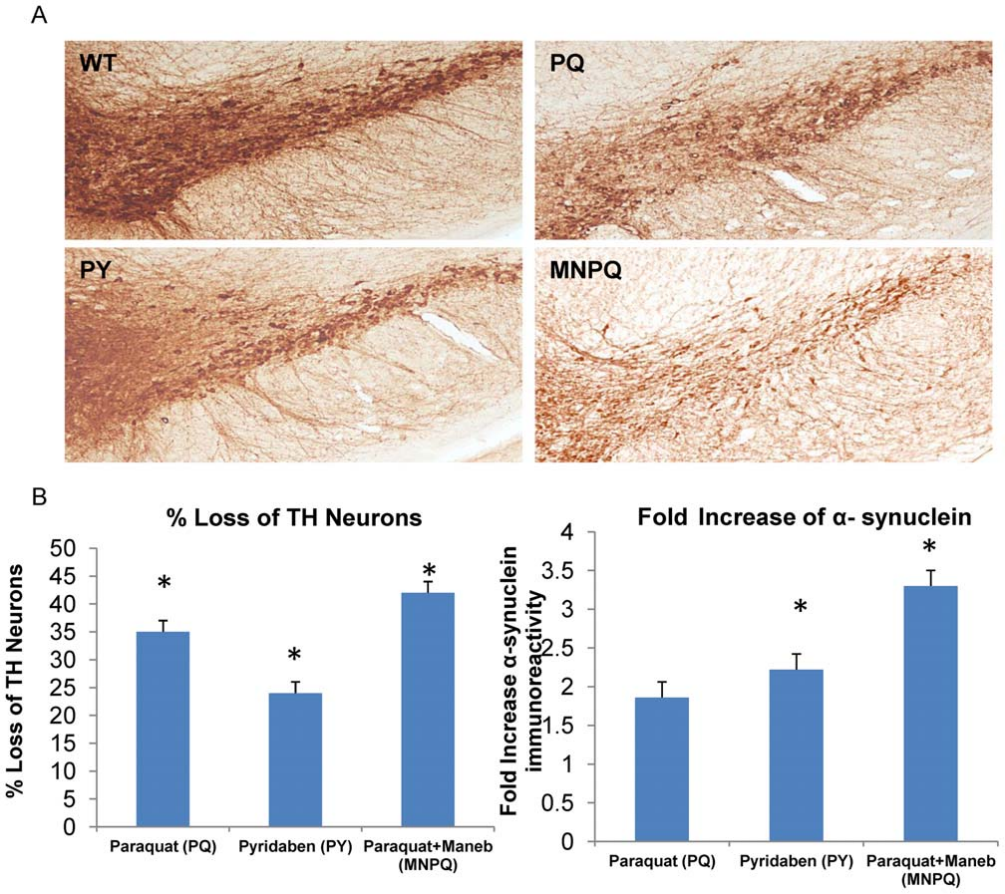
Pesticide induced loss of TH+ neurons and increased α-synuclein immunoreactivity. A) Immunostaining of SN of PQ, PY, MNPQ and wild type (WT) mice with tyrosine hydroxylase antibody. B) Percent loss of TH+ neurons and fold increase of α-synuclein immunoreactivity compared to wild type control mice.

Tyrosine hydroxylase (TH) immunostaining showed a significant loss of TH+ neurons in the SN of PQ, PY and MNPQ exposed mouse models (Figure 1A). The percent loss of TH+ neurons in SN of PQ, PY and MNPQ mouse models were significantly less than controls by 35%, 24% and 42% respectively at p = 0.05. The fold increase of α-synuclein aggregates in PQ mouse model was 1.8 which was not significant, while in the PY and MNPQ models the alpha-synuclein increased by 2.2 and 7.1 fold respectively, which were significant (p = 0.05) (Figure 1B). Therefore, the administration of pesticides PQ, PY and MNPQ using subcutaneous alzet pump for one week was able to model PD, by eliciting a significant loss of TH+ neurons in the SN along with an increase in α-synuclein immunoreactivity.

### PQ, PY and MNPQ Induce Differential Gene Expression in VMB and STR

**Figure 2:**
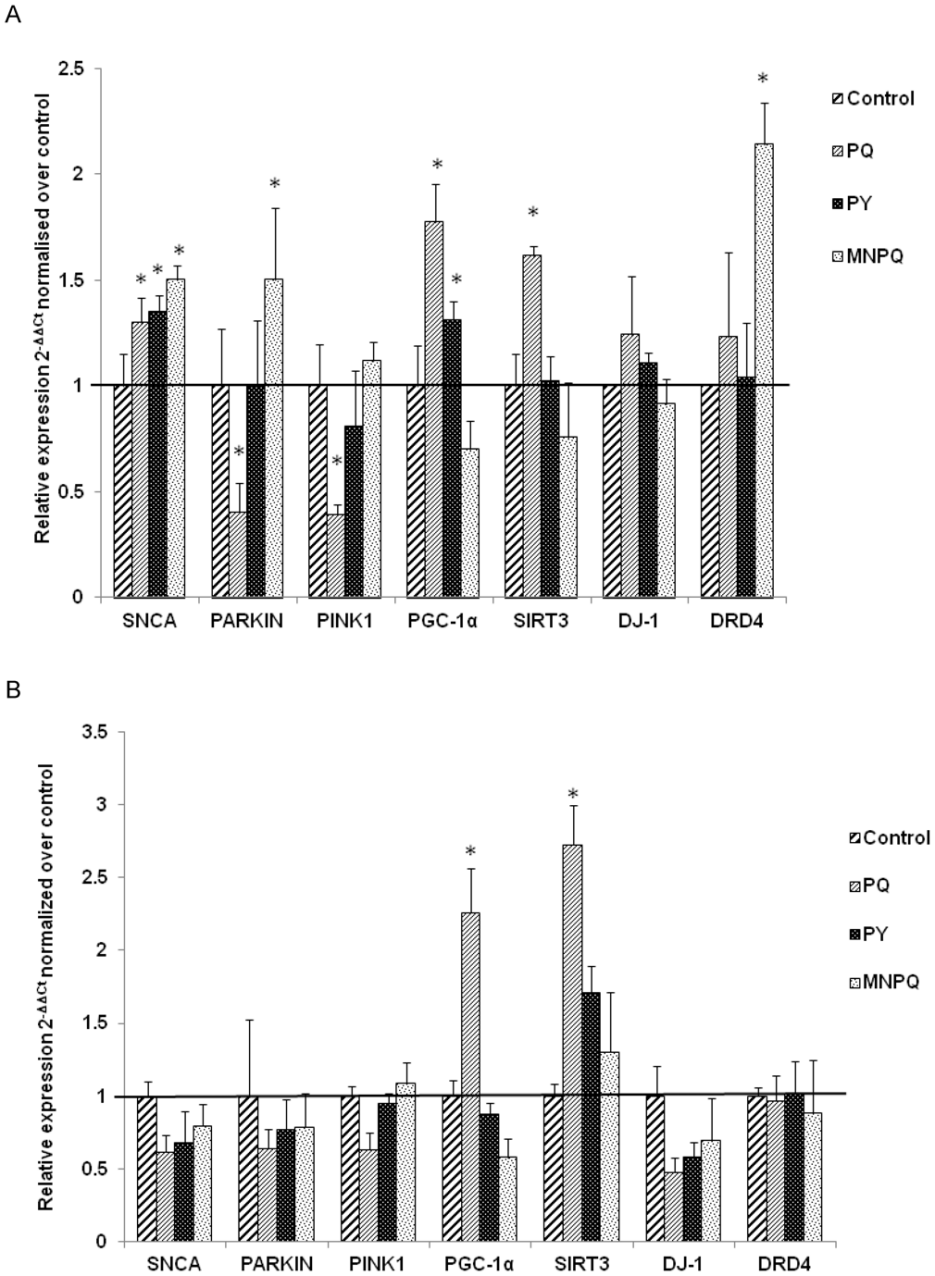
Validation of RNA-Seq gene expression fold changes by Taqman real time PCR. A) Gene expression fold changes in VMB. B) Gene expression fold changes in the STR.

Since we had a moderate but not complete loss of TH+ neurons, we examined whether we could detect early signaling events by monitoring mRNA expression changes by transcriptome sequencing. Differential gene expression in the VMB and the STR in the PQ, PY and MNPQ models was observed with RNA-Sequencing. RNA-Seq gene expression raw data from VMB and STR from PQ, PY, MNPQ and control samples have been deposited in NCBI's Gene Expression Omnibus (GEO) and are accessible through GEO Series accession number GSE36232 (http://www.ncbi.nlm.nih.gov/geo/query/acc.cgi?acc=GSE36232). Average fold changes across replicates were determined and compared to wild type control mice for every gene and/or isoform in a reference sequence (RefSeq) collection. The RNA-Seq gene expression fold changes were validated for seven PD genes by Taqman assays (Table 1). Gene expression fold changes in VMB and STR (Figure 2A and Figure 2B respectively) were determined using the 2^−ΔΔCt^ method as compared to 18S ribosomal RNA as a housekeeping gene, and normalized to controls. When the gene expression fold changes were less than 0.3 different as determined by RNA-Seq or Taqman assay and in the same direction we considered them concordant. There was a seventy one percent concordance between gene expression fold changes as determined by RNA-Seq and Taqman assays.

**Table 1:** Taqman assays used for quantitative RT-PCR.

Taqman Assay ID	Gene Symbol	Description
Mm01208835_m1	PPARGC1A	Peroxisome proliferator-activated receptor gamma coactivator 1-alpha (PGC-1α)
Mm01188700_m1	PARK1	Alpha-synuclein (SNCA)
Mm00450186_m1	PARK2	E3 ubiquitin-protein ligase (PARKIN)
Mm00550827_m1	PARK6	PTEN induced putative kinase 1 (PINK1)
Mm00498538_m1	PARK7	DJ-1
Mm00432893_m1	DRD4	D(4) dopamine receptor
Mm00452129_m1	SIRT3	NAD-dependent deacetylase sirtuin-3
Hs99999901_s1	RN18S	18S Ribosomal RNA

**Figure 3:**
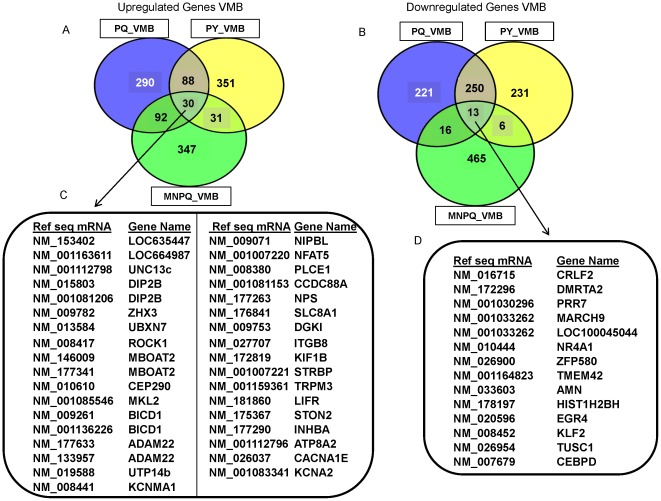
Common genes in the VMB between PQ, PY and MNPQ mouse models of PD. A) Venn diagram of 500 up-regulated VMB transcripts from PQ, PY and MNPQ models of PD. B) Venn diagram of 500 down-regulated VMB transcripts from PQ, PY and MNPQ models of PD. C) Common up-regulated genes in the VMB of PQ, PY and MNPQ models of PD. D) Common down-regulated genes in the VMB of PQ, PY and MNPQ models of PD.

**Figure 4:**
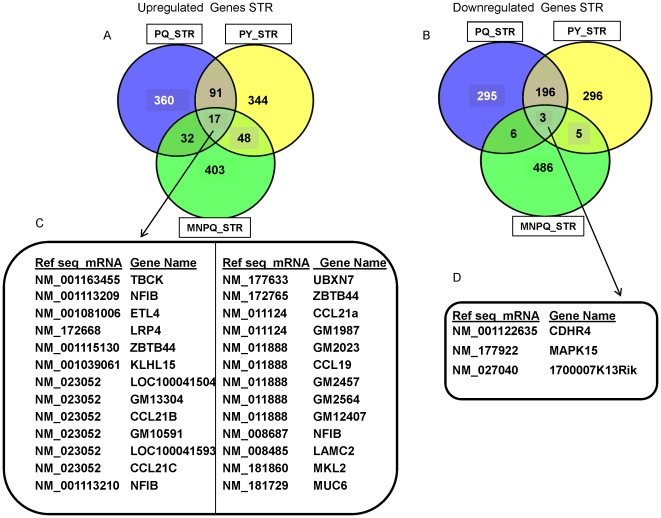
Common genes in the STR between PQ, PY and MNPQ mouse models of PD. A) Venn diagram of 500 up-regulated STR transcripts from PQ, PY and MNPQ models of PD. B) Venn diagram of 500 down-regulated STR transcripts from PQ, PY and MNPQ models of PD. C) Common up-regulated genes in the STR of PQ, PY and MNPQ models of PD. D) Common down-regulated genes in the STR of PQ, PY and MNPQ models of PD.

To compare the common transcripts between pesticides we used the web based tool venny (http://bioinfogp.cnb.csic.es/tools/venny/index.html). We compared the 500 up- and down-regulated transcripts in the PQ, PY and MNPQ models to link the common genes in our study to the most significant pathways previously identified in PD. The common up- and down-regulated transcripts in the VMB are depicted in Figure 3A and Figure 3B respectively, and their gene identifiers provided in Figure 3C and Figure 3D respectively. The common up- and down-regulated transcripts in STR are depicted in Figure 4A and Figure 4B respectively, and their gene identifiers provided in Figure 4C and Figure 4D respectively. We further describe some of the most interesting genes in relationship to possible involvement in PD pathways. The up-regulated VMB transcripts *Slc8a1*, *Plce1* and *Cacna1e* belong to calcium signaling pathways. These changes in calcium signaling may be important in relationship to PD pathogenesis [22]. PD patients taking calcium channel blockers show a twenty seven percent reduction in risk of developing PD compared to those not using these drugs [22]. Reducing Ca^2+^ influx into SN DA neurons increases the resistance of these neurons to toxins which are used to generate animal models of PD [23].

Some of the other up-regulated transcripts in the VMB include members of axon guidance signaling (*Rock1*, *Adam22*), zinc finger proteins (*Eif2c3* and novel KRAB box and zinc finger, C2H2 type domain containing protein) and one of the candidate genes for late onset Alzheimer's Disease (*Plce1*) [24]. *Nps* (neuropeptide S) stimulates dopaminergic neurotransmission [25], *MBoat2* belonging to MBOAT family of multispanning transmembrane enzymes, catalyze the acylation of secreted signalling proteins of the Hedgehog, Wnt and ghrelin families [26].

Some of the significant genes in our study (Table S1 and Table S2) have been linked to PD. *Nrtn* (neurturin precursor) expression was down-regulated in the VMB of PQ and MNPQ mouse models. NRTN belongs to the glial cell line-derived family of neurotrophic factors, which have been recognized to have the potential to repair damaged and degenerating neurons and restore their function. Interestingly, delivery of *Nrtn* using adeno associated viral (AAV) vectors has been tested in human PD, and nonclinical translational experiments to test the safety, feasibility and effectiveness of targeting the SN with AAV2-*Nrtn* to support clinical studies to enhance the trophic response in PD DA neurons [27]. *Phox2a* (paired mesoderm homeobox protein 2A) expression was down-regulated in the VMB of PY mouse model. *Phox2a* along with *Lmx1b* (LIM-homeodomain protein) are part of a novel gene regulatory network that controls the spatiotemporal generation of DA neurons during midbrain development [28]. Another gene down-regulated in the VMB of PY model is, urocortin precursor (*Ucn*), a group of peptides, closely related to corticotrophin-releasing factor (CRF). *Ucn* has been reported to arrest the progression of or even reverse nigral lesions in the rat 6-hydroxydopamine (6-OHDA) and lipopolysaccharide (LPS) models of PD [29]. *Bcl2a1* (B-cell lymphoma 2-related protein A1) is up-regulated in the VMB of PY model in our study. *Bcl2a1* is a highly regulated nuclear factor κB (NF-κB) target gene that has important pro-survival functions [30]. Vitamin D induced *Camp* gene up-regulated in VMB of PY model encodes cathelicidin antimicrobial peptide, which initiates the innate immune responses to bacterial infection, and can act as signaling molecule to regulate immune system function [31].

Delta-aminolevulinate synthase 2 (*Alas2*) was up-regulated in the STR of MNPQ mouse model. This gene encodes a protein that catalyzes the first step in the heme biosynthetic pathway. The expression of *Alas2* along with *Fech* and *Blverb*, the other heme metabolism genes is tightly correlated with α-synuclein expression in human PD [32]. This correlates with MNPQ mouse model having the highest fold increase of α-synuclein in our study. Human serum amyloid A (*Saa*), a high-density lipoprotein-associated lipoprotein upregulated in VMB of MNPQ mouse model is known to modulate inflammation and the metabolism and transport of cholesterol [33]. Dysregulation of ion channels are known to cause pathology in PD [34]. Ion channel genes which ranked high in our gene list included ionotropic glutamate receptor genes (*Grin2a*, *Grin2b*), ligand gated chloride channel gene (*Gabra6*) and potassium voltage gated channel genes (*Kcnh7*). *Grin2a* is also a PD disease modifier gene via an interaction with coffee [35]. *Ryr2* (Ryanodine receptor 2, cardiac) was up-regulated in the STR of PQ model in our study. An up-regulation of *Ryr2* receptors with paraxanthine (PX; 1,7-dimethylxanthine), the primary metabolite of coffee was found to be protective against MPP+ *in vitro* [36]. The top ranking ten up-regulated and down-regulated genes of PQ, PY and MNPQ datasets identified by IPA in the VMB and STR are listed in Table S1 and Table S2 respectively.

In summary, we found several differentially expressed genes that are related to PD or other neurological diseases, we therefore decided to perform a systematic analysis to look for cell signaling and metabolic pathways, and gene networks to identify potential targets for therapeutic intervention.

### Concordant Signaling Pathways in the VMB and STR of PQ, PY and MNPQ Mouse Models

**Figure 5:**
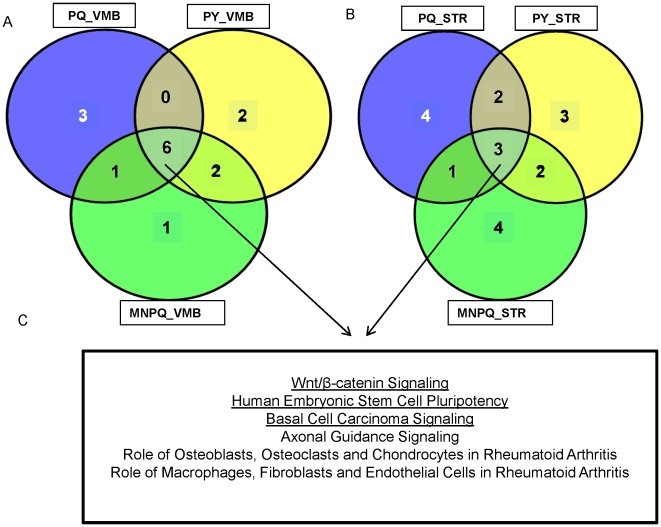
Venn diagram of top ten signaling pathways in PQ, PY and MNPQ models of PD. A) Signaling pathways in VMB. B) Signaling pathways in STR. C) Common overlapping pathways in VMB and STR are underlined. Considering the top ten canonical pathways there are six pathways common in VMB of PQ, PY and MNPQ mouse models. There are three pathways common in STR of PQ, PY and MNPQ mouse models. All the three common canonical pathways in STR overlap with pathways common in the VMB between the three pesticide models.

In order to find enriched signaling pathways in PQ, PY and MNPQ mouse models from the VMB and STR, IPA was employed. All differentially expressed genes with ≥1.5 or ≤1.5 fold changes were uploaded for analysis. The top ten canonical pathways in the VMB of PQ, PY and MNPQ models of PD along with associated molecules are listed in Table S3, Table S4 and Table S5 respectively. We found pathways previously implicated in human PD such as such as axonal guidance signaling [17], [37], pathways linked to other neurodegenerative diseases such as Wnt/β catenin signaling [38], in addition to pathways not previously associated with PD such as basal cell carcinoma, role of osteoblasts, osteoclasts and chondrocytes in rheumatoid arthritis, human embryonic stem cell pluripotency, role of macrophages, fibroblasts and endothelial cells in rheumatoid arthritis. Alterations in Wnt signaling are associated with neurodegenerative diseases while overactivation of Wnt signaling is a common theme in several types of human tumors [38]. The top ten canonical pathways in the STR of PQ, PY and MNPQ models of PD are listed in Table S6, Table S7 and Table S8 respectively. Considering the top ten pathways in the three pesticide models, there were six concordant pathways in the VMB (Figure 5A) and three concordant pathways in the STR (Figure 5B). The three concordant STR pathways that overlapped with the VMB pathways were Wnt/β-catenin signaling, human embryonic stem cell pluripotency and basal cell carcinoma signaling (Figure 5C).

Some of the significant canonical pathways in the VMB and STR of PQ, PY and MNPQ mouse models were common with pathways observed in human PD (Table 2 and Table 3) [18]. Therefore, axonal guidance signaling and wnt/β catenin signaling, and their associated genes are not only common between individual pesticide models, but also overlap with human PD.

**Table 2:** Overlapping signaling pathways between human PD transcriptome studies and signaling pathways in VMB of PQ, PY and MNPQ mouse models of PD.

IPA Pathway Category	Ventral mid brain (VMB) pathways common with human								
	SN [17]	BA9 [17]	PU [17]	SFG [14]	L SN [14]	M SN [14]	SN [37]	OC[15]	PU [15]
Amyotrophic Lateral Sclerosis Signaling				**x**				**x**	
Glutamate Receptor Signaling				**x**	**x**				
Calcium Signaling		**x**						**x**	**x**
Actin Cytoskeleton Signaling				**x**		**x**	**x**		
PTEN Signaling		**x**		**x**	**x**	**x**			
LXR/RXR Activation	**x**								
PPAR Signaling	**x**	**x**				**x**	**x**		
PPARα/RXRα Activation	**x**								
ERK/MAPK Signaling		**x**		**x**				**x**	
Axonal Guidance Signaling		**x**			**x**		**x**	**x**	
IL-6 Signaling	**x**	**x**							
Ephrin Receptor Signaling	**x**	**x**		**x**		**x**			
Erythropoietin Signaling								**x**	
G-Protein Coupled Receptor Signaling			**x**		**x**	**x**		**x**	
RAR Activation		**x**					**x**	**x**	
IGF-1 Signaling	**x**	**x**		**x**		**x**	**x**	**x**	
JAK/Stat Signaling	**x**	**x**		**x**		**x**	**x**		
Death Receptor Signaling		**x**							
Aryl Hydrocarbon Receptor Signaling							**x**	**x**	
PI3K/AKT Signaling	**x**	**x**		**x**			**x**		
Hepatic Cholestasis	**x**								
IL-10 Signaling	**x**								

All human IPA analysis were performed by [18].

**x** = Common Pathways.

SN: Substantia nigra, BA9: Brodman area 9, SFG: Superior frontal gyrus, L SN: Lateral substantia nigra, M SN: Medial substantia nigra, PU: Putamen, OC: Occipital cortex.

**Table 3:** Overlapping signaling pathways between human PD transcriptome studies and signaling pathways in STR of PQ, PY and MNPQ mouse models of PD.

IPA Pathway category	Striatum (STR) pathways common with human								
	SN [17]	BA9 [17]	PU [17]	SFG [14]	L SN [14]	M SN [14]	SN [37]	OC [15]	PU [15]
Calcium Signaling		**x**						**x**	**x**
PTEN Signaling		**x**		**x**	**x**	**x**	**x**		
Amyotrophic Lateral Sclerosis Signaling				**x**				**x**	
Axonal Guidance Signaling		**x**			**x**		**x**	**x**	
IL-6 Signaling	**x**	**x**							
G-Protein Coupled Receptor Signaling			**x**		**x**	**x**		**x**	
Hepatic Cholestasis	**x**								
PPARα/RXRα Activation	**x**								
Cardiac β-adrenergic Signaling		**x**			**x**				
Estrogen Receptor Signaling						**x**	**x**	**x**	
Wnt/β-catenin Signaling		**x**							
Serotonin Receptor Signaling									**x**
LXR/RXR Activation	**x**								

All human IPA analysis were performed by [18].

**x** = Common Pathways.

SN: Substantia nigra, BA9: Brodman area 9, SFG: Superior frontal gyrus, L SN: Lateral substantia nigra, M SN: Medial substantia nigra, PU: Putamen, OC: Occipital cortex.

### Common Genes from Network Analysis in Pesticide Models of PD

**Figure 6:**
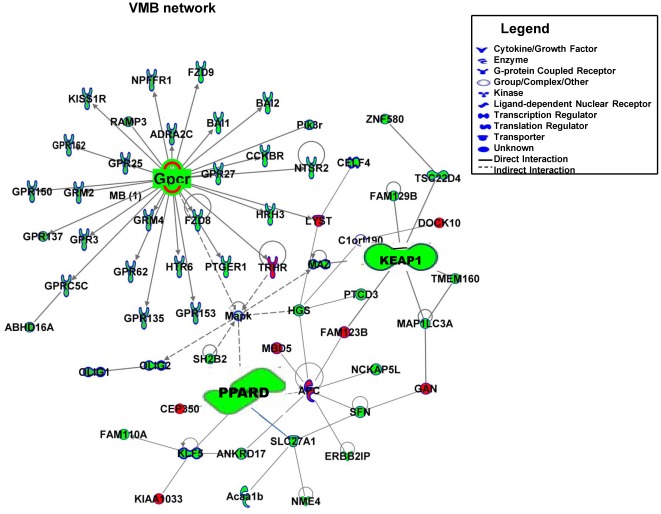
Global gene network in VMB of PQ, PY and MNPQ mouse models of PD. The genes from the most significant network of each individual pesticide mouse model were overlayed to generate global gene networks in the VMB. Down-regulated genes are highlighted in green, up-regulated genes are highlighted in red. The focus molecules-G protein coupled receptors (GPCRs) members, Kelch-like ECH-associated protein 1 (*Keap1*) and peroxisome proliferator-activated receptor delta (*Pparδ*) are down-regulated in VMB.

**Figure 7:**
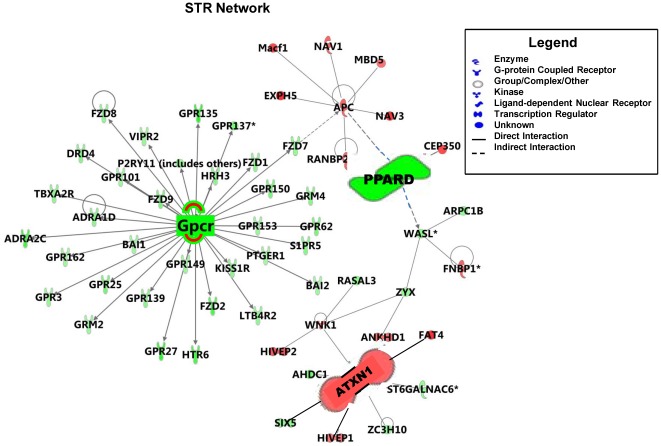
Global gene network in STR of PQ, PY and MNPQ mouse models of PD. The genes from the most significant network of each individual pesticide mouse model were overlayed to generate global gene networks in the STR. Down-regulated genes are highlighted in green, up-regulated genes are highlighted in red. The focus molecules- G protein coupled receptors (GPCRs) members and *Pparδ* are down-regulated while *Atxn1* is up-regulated.

The genes from the most significant network of each individual pesticide were used to generate global gene networks in the VMB (Figure 6) and the STR (Figure 7). Red indicates up-regulated genes, green indicates down-regulated genes and grey indicate no change in gene expression. The higher the intensity of the color, higher the gene expression fold-change. Network representation allows investigation of functional associations of commonly expressed genes following exposure to PQ, PY and MNPQ. The key genes in the VMB network are *Keap1* (kelch-like ECH-associating protein 1), *Pparδ* (peroxisome proliferator-activated receptor delta), MAPKs (mitogen activated protein kinases) and several GPCRs (G protein coupled receptors), are all down-regulated. The key genes in the STR network are *Atxn1* (ataxin 1) which is up-regulated, while *Pparδ* and several GPCRs are down-regulated. *Pparδ* and GPCRs are common in both VMB and STR, indicating that they could be therapeutic targets.

The common GPCR's in VMB and STR are *Gpr101*, *Gpr135*, *Gpr,150*, *Gpr153*, *Gpr162*, *Gpr25*, *Gpr27*, *Gpr3*, *Gpr62*, metabotropic glutamate receptors (*Grm2* and *Grm4*), cholecystokinin B receptor (*Cckbr*), chemokine C receptor 4 (*Ccr4*), histamine receptor (*Hrh3* and *Hrh6*), Kiss1 receptor (*Kiss1r*) and prostaglandin E receptor 1 (*Ptger1*). The other common molecule in the VMB and STR is α-2 adrenergic receptor (*Adra2c*) which has been associated with PD [39]. These findings therefore show that pesticides alter gene expression in networks, some of which have been implicated in the pathogenesis of PD.

## Discussion

In our study, administration of pesticides produced the neuropathological features of PD, in that they produced loss of nigrostriatal DA neurons and increased α-synuclein immunoreactivity, which is a protein found in lewy bodies, and is the most characteristic feature of idiopathic PD. Our mouse models of PQ, PY and MNPQ exposure produced 35%, 24% and 42% loss of nigrostriatal neurons, allowing us to examine RNA changes in circumstances in which the observed alterations are not merely a reflection of cell loss and gliosis. Our results are the first to show that PY exposure in mice can model PD, in that it produces a significant loss of TH+ neurons in the SN and α-synuclein aggregates. This is the first comprehensive evaluation using RNA-Seq of the transcriptome of brains from PQ, PY and MNPQ treated mice. Our PQ and MNPQ models are similar to previous models in that we observed a loss of TH+ neurons and increased α-synuclein aggregates. The fold increase of α-synuclein aggregates in the PQ mouse model was 1.8 which was not significant in our study. Paraquat induced upregulation of α-synuclein as assessed by immunohistochemistry has also been reported in previous PQ models of PD, although the stereological technique for quantification of dopaminergic neurons was not performed by any of the groups. We observed an exacerbation of toxicity with the combination of PQ and MN as previously reported [40]. In our experiments, we administered the pesticides using alzet pumps, which have the advantage of producing a more even exposure, which is more likely to mimic human exposure than the pulsatile effects of i.p. injections.

The genes identified by network analysis in both the VMB and STR are, peroxisome proliferator activated receptor delta (*Pparδ*) and G protein coupled receptors (GPCRs). While *Keap1* and MAPK's are specific to VMB, *Atxn1* is specific to STR. Evidence for a role of GPCRs in PD comes from *in vitro* studies and animal models supporting the use of agonists of Group III metabotropic glutamate (mGlu) receptors belonging to the GPCR family, as potentially important drug targets for providing beneficial symptomatic effects [41]. Therefore, group III mGlu receptors are promising targets for drug discovery in PD.

PPARδ regulates the expression of peroxisome proliferator-activated receptor gamma coactivator 1-alpha (*Pgc-1α*). This is of particular importance due to a recent meta-analysis of microarray gene expression studies of PD postmortem brain tissue which showed that PGC-1α expression is significantly reduced [42]. Furthermore, PARKIN deficiency results in increased levels of the protein PARIS which binds to the PGC-1α promoter, and reduces its expression, and producing loss of dopaminergic neurons [43]. PGC-1α regulates the antioxidant enzymes, Cu/Zn superoxide dismutase (*Sod1*), and manganese superoxide dismutase (*Sod2*) and glutathione peroxidase (*Gpx1*) [44] and also controls mitochondrial biogenesis [45]. PPARδ and PGC-1α cooperatively induce heme oxygenase-1 (*Ho-1*) called *Hmox1* in mice [46]. The nuclear translocation of HO-1 also referred to as heat shock protein 32 (HSP-32) takes place under stress conditions and leads to the loss of its activity. This inactive nuclear form of HO-1 influences the expression of cytoprotective genes [47]. Impairment of HO-1 production following oxidative stress accelerates the neurodegeneration in cellular models with mutant PINK1 [48]. Since, there is evidence for a deficiency of PGC-1α in PD [42] and there is evidence of mitochondrial dysfunction and increased oxidative damage in PD [9], a deficiency of PPARδ may play an important role in PD pathogenesis.

Oxidative stress has been associated with the etiology of both sporadic and monogenic forms of PD. Therefore, the down-regulation of *Keap1* in VMB in our study is significant because the *Nrf2-Keap1* pathway orchestrates protection of cells against oxidative stress by up-regulating expression of nuclear cytoprotective genes. This classic adaptation has been designated the environmental stress response (ESR) [49]. KEAP1 acts as a stress sensor, whereas NRF2 serves as the effector of the ESR. Besides, KEAP1 also senses the intracellular concentrations of NO (nitric oxide), Zn^2+^, and alkenals, which collectively serve as second messengers that may signify danger and/or damage [49]. When the KEAP1 sensor is down-regulated, the effector NRF2 may be ineffective in inducing cytoprotective genes.

*Dj-1*, is one of the genes responsible for early onset autosomal recessive PD. DJ-1, the protein product of *Dj-1*, stabilizes NRF2 by preventing its association with the inhibitor protein KEAP1, with subsequent ubiquitination and degradation of NRF2 [50]. Therefore, the down-regulation of both *Keap1* and *Dj-1* in our study may be responsible for the failure of an up-regulation of the *Nrf2* target cytoprotective genes. The expression of several human PD genes was down-regulated in our study including *Uch-l1*, *Pink1*, *Atp13a2*, *Omi/Htra2*, *Pla2g6* in VMB and STR in all the three pesticide models. Several of these genes are involved in mitochondrial dynamics and turnover [51], as well as ubiquitination of aberrantly misfolded proteins.

The ubiquitin proteosome system (UPS) plays a critical role in PD pathogenesis. Three PD-related proteins PARKIN, PINK1 and DJ-1 physically interact and form a functional novel E3 ligase complex called PPD to regulate UPS mediated protein degradation [52]. Evidence for UPS involvement in response to pesticide exposure comes from the up-regulated genes in our study, *Atxn1* and *Sacs*, and down-regulated heat shock protein 70 (*Hsp70*) gene. SACSIN, the protein product of the *Sacs* gene, one of the top ranking genes in our study, is a coregulator of chaperones and prevents polyglutamine mediated aggregation of *Atxn1* [53]. The role of *Atxn1* is unknown in PD, whereas SACSIN has been classified as a molecular chaperone which cooperates with members of the HSP70 chaperone [54]. There was a down-regulation of heat shock protein 70 transcripts *Hspa1a* and *Hspa1b* in our study. Wild type and mutant α-synuclein are degraded by chaperone mediated autophagy (CMA) [55]. HSP70 keeps α-synuclein soluble and promotes α-synuclein degradation via CMA and/or the UPS, preventing toxicity [56]. We previously showed that induction of HSP70 with celastrol produces protective effects in the MPTP model of PD [57].

When comparing the same brain region between the PQ, PY and MNPQ models, we found several concordant signaling pathways which concur with human PD pathways identified in independent gene expression profiling studies [18]. The Wnt signaling pathway was a top concordant pathway in our study in the VMB and STR. Wnt signaling is critically involved in key processes for the formation and plasticity of the nervous system, neurogenesis, axon guidance, dendritic development, synaptic differentiation and plasticity [38]. Several WNTs are expressed in the VMB where they regulate the birth of DA neurons [58] and play an important role in dopamine related adult brain functions [59]. Abnormalities of Wnt signaling are implicated in AD [38], PD [60], [61], schizophrenia [62], and frontotemporal dementia [63]. In our study, we observed a down-regulation of the Wnt gene expression in VMB and STR including *Wnt1, Wnt2, Wnt3, Wnt4, Wnt5a, Wnt6, Wnt7a, Wnt7b, Wnt10a and Wnt10*. Therefore, up-regulation of Wnt gene expression may be an effective therapeutic strategy for PD. A previous study found that *Wnt5a*-treated neural stem cells are an efficient and safe source of DA neurons for cell replacement therapy in PD, due to improved differentiation and functional integration of stem cell–derived DA neurons *in vivo* [60]. Since we observed that *Wnt5a* signaling was impaired in pesticide treated mice, this may contribute to impaired recovery of dopaminergic neurons.

Embryonic stem cell pluripotency signaling was concordant in the VMB and STR between the three pesticides induced PD mouse models in our study. Wnt signaling maintains pluripotency in embryonic stem cells [38]. Axonal guidance signaling which was concordant in the VMB in our study is in agreement with human PD studies [37] as well as in human neuroblastoma cell line treated with PQ [64]. During development, precise temporal and spatial gradients are responsible for guiding axons to their appropriate targets. WNTs are morphogens that have been identified as axon guidance molecules. The canonical pathway - role of macrophages, fibroblasts and endothelial cells in rheumatoid arthritis (RA) suggests a relationship between chronic inflammation and PD. Basal cell carcinoma signaling is one of the pathways in our study, although its relationship to PD is not yet known. The possible link between PD and basal cell carcinoma cannot be ruled out in light of the finding that several PD genes such as *Parkin*, *Pink1* and *Dj-1* have been associated with certain cancers [65].

In conclusion, PQ, PY and MNPQ elicit concordant signaling pathways in the VMB and STR in mouse models which overlap with some pathways observed in human PD, suggesting that similar mechanisms may occur in both. Common focus molecules in the VMB and STR identified by network analysis and pathway analysis may be used to identify targets for therapeutic intervention.

## Materials and Methods

### Ethics Statement

All experiments were conducted within National Institutes of Health guidelines for animal research and were approved by the Weill Cornell Medical College Animal Care and Use Committee under animal protocol number 2010-0043. The mice were kept on a 12-hr light/dark cycle, with food and water available *ad libitum*. All surgeries were performed under isoflurane anesthesia and all efforts were made to minimize animal suffering

### Mice and pesticide paradigm

Three month old C57Bl6 male mice (Jackson Labs, Bar Harbor, Maine, USA) were administered PQ, PY and MNPQ of pestanal grade (Sigma Inc, MO, USA) using subcutaneous alzet pumps (Durect Corporation, Cupertino, CA, USA) for 7 days. Concentrations were PQ (10 mg/kg/day), PY (3.5 mg/kg/day) and a combination of PQ (10 mg/kg/day)+MN (30 mg/kg/day). Control C57Bl6 mice were administered PBS mixed with 1∶8 mix of ethanol (Sigma-Aldrich, St Louis, MO, USA) and neobee® M-5 oil (Stepan Company, Northfield, IL, USA) as vehicle. Six mice were treated per group. PQ was dissolved in 1× phosphate buffered saline (Mediatech Inc., Manassas, VA, USA) and PY and MN were dissolved in a 1∶8 mix of ethanol and neobee® M-5 oil. The vehicle for administration of all pesticides was PBS mixed with a 1∶8 (ethanol: neobee oil) mixture. Mice were sacrificed at day 7 and their brains were harvested for immunohistochemistry and RNA-Sequencing.

### Immunohistochemistry

Free-floating brain sections were prepared and processed for immunohistochemistry using a modified avidin-biotin-peroxidase technique. Briefly, the sections were pretreated with 3% H_2_O_2_ in 0.1 M sodium phosphate-buffered saline (PBS) for 30 min. The sections were rinsed in PBS twice for 5 min each. The sections were incubated sequentially in (a) 1% bovine serum albumin (BSA)/0.2% Triton X-100 (Sigma, St. Louis, MO) for 30 minutes, (b) primary antibody (diluted in PBS/0.5% BSA) for 18 hours, (c) appropriate biotinylated IgG (1∶200, diluted in PBS/0.5% BSA, Vector Laboratories, Burlingame, CA) for 1 hour, and (d) avidin-biotin-peroxidase complex (1∶200 in PBS; Vector Laboratories) for 1 hour. The immunoreactions were visualized using 3,3′-diaminobenzidine tetrahydrochloride dihydrate (DAB) as the chromogen. All incubations and rinses were performed at room temperature with agitation using an orbital shaker. The sections were mounted onto gelatin-coated slides, dehydrated, cleared in xylene and coverslipped. The primary antibodies used were rabbit anti-tyrosine hydroxylase (TH) affinity purified antibody (1∶4,000; Chemicon, Temecula, CA, USA) and mouse anti-α-synuclein (1∶1,000, BD Transduction, Chicago, IL, USA). The total numbers of TH positive neurons were counted using a stereological technique (optical fractionator) from the Stereo Investigator software v. 9.12 (Microbrightfield, Burlington, VT). A series of seven cryosections (50-µm thick) through the SN per mouse was analyzed. For quantitation of α-synuclein immunoreactive cells in the SN, sections adjacent to those used for counting TH were used and counted manually. Seven sections encompassing the entire SN and containing the SN were analyzed.

### Library preparation and RNA-Sequencing

RNA-Sequencing was carried out for three replicates per pesticide per brain region. The right half of the mouse brain was used for dissecting out the VMB and STR for RNA isolation using Trizol Reagent (Invitrogen, Carlsbad, California,USA). RNA was purified using RNA clean and concentrator (Zymo Research Corporation, Irvine, CA, USA). RNA was quantified using the Agilent 2100 Bioanalyzer (Agilent Technologies, Santa Clara, CA, USA). One microgram of total RNA with a RNA Integrity Number (RIN) more than eight was used for library preparation using mRNA-Seq sample preparation kit (Illumina, Inc., San Diego, CA, USA). RNA-Sequencing was done using an Illumina Genome Analyzer.

### Analysis of RNA-Seq data

After mRNA library quality check using the Bioanalyzer, polyadenylated transcriptome sequencing was performed following the Illumina protocol using the GAIIx sequencer with 40 cycles of incorporation in single end mode. 20–30 M short reads were obtained for each experiment and were aligned to RefSeq transcripts (June 2010 release) using BWA with default parameters [66]. All RNA-Seq had >70% mappable reads. After alignment, transcript levels were then estimated and normalized using the RPKM approach [67]. Differential expression was analyzed using unpaired t-tests and the Benjamini-Hochberg correction was applied to control the false discovery rate. All statistical analysis was performed using the R statistical software. The data discussed in this publication has been deposited in NCBI's Gene Expression Omnibus and are accessible through GEO Series accession number GSE36232 (http://www.ncbi.nlm.nih.gov/geo/query/acc.cgi?acc=GSE36232).

### Quantitative real time PCR

Gene expression for seven PD related genes were further validated using quantitative reverse transcriptase real time (RT) PCR. cDNA was prepared from 500 ng of total RNA with the same RNA samples used for RNA-Sequencing experiments using superscript III first strand synthesis system for RT-PCR (Invitrogen, Carlsbad, California, USA). Quantitative RT-PCR was performed using Taqman gene expression master mix and Taqman primer probe mix in a 8 µL reaction mix in 7900 HT Fast Real time PCR system (Applied Biosystems, Carlsbad, California, USA) using Taqman assays (Table 1). The house keeping gene was 18S ribosomal RNA. Fold changes were calculated using the 2^−ΔΔCt^ method and plotted as fold differences of relative gene expression normalized to controls.

### Pathway Analysis

All gene sets were subject to ingenuity pathway analysis (IPA, Ingenuity Systems, www.ingenuity.com) in order to identify significantly deregulated canonical pathways and to generate networks of deregulated genes. To do this, the gene expression data sets were uploaded into the application. All genes with ≥1.5 or ≤1.5 gene expression fold changes were selected for uploading. Each gene identifier was mapped to its corresponding gene object in the Ingenuity Pathways knowledge base. On the basis of the IPA library, canonical pathway analyses identified pathways that were most significant in the data sets. To generate networks, the genes from the data set were overlaid onto a global molecular network developed from information contained in the Knowledge Base. Gene networks were then algorithmically generated on the basis of their connectivity. The significance of the association between the datasets and the canonical pathways were measured using the Fischer's exact test, to calculate the probability that the association between the gene list and the canonical pathway was explained by chance alone and calculating the ratio of the number of genes from the gene list that mapped to the pathway divided by the total number of molecules that exist in the canonical pathway. Pathways with a high ratio and a low P-value are indicative of potentially good candidates for further exploration. P Values were represented as −log(p value) and all values above 1.3 are significant.

## Supporting Information

Table S1 **Top genes and their expression levels in VMB of paraquat, pyridaben and maneb+paraquat mouse models of PD.**
(XLS)

Table S2 **Top genes and their expression levels in STR of paraquat, pyridaben and maneb+paraquat mouse models of PD.**
(XLS)

Table S3 **Top ten canonical pathways in VMB of paraquat mouse model of PD**
(XLS)

Table S4 **Top ten canonical pathways in VMB of pyridaben mouse model of PD**
(XLS)

Table S5 **Top ten canonical pathways in VMB of maneb+paraquat mouse model of PD**
(XLS)

Table S6 **Top ten canonical pathways in STR of paraquat mouse model of PD**
(XLS)

Table S7 **Top ten canonical pathways in STR of pyridaben mouse model of PD**
(XLS)

Table S8 **Top ten canonical pathways in STR of maneb+paraquat mouse model of PD.**
(XLS)
